# Genomic Insights Into Species Delimitation and the Evolutionary History of Mimetic *Aletis* Moths (Lepidoptera, Geometridae) in the Afrotropics

**DOI:** 10.1002/ece3.72745

**Published:** 2025-12-18

**Authors:** Kyung Min Lee, Hermann Staude, Sille Holm, Elina Laiho, Leidys Murillo‐Ramos, Øystein H. Opedal, Hossein Rajaei, Reinier F. Terblanche, Magda Botha, Pasi Sihvonen

**Affiliations:** ^1^ Zoology Unit, Finnish Museum of Natural History University of Helsinki Helsinki Finland; ^2^ Caterpillar Rearing Group, LepSoc Africa Magaliesburg South Africa; ^3^ Department of Ecology Swedish University of Agricultural Sciences Uppsala Sweden; ^4^ Department of Biology Universidad de Sucre Sincelejo Colombia; ^5^ Department of Biology, Division of Biodiversity & Evolution Lund University Lund Sweden; ^6^ State Museum of Natural History Stuttgart Stuttgart Germany; ^7^ Unit for Environmental Sciences and Management North West University Potchefstroom South Africa

**Keywords:** allopatry, caterpillar, ddRAD‐sequencing, gene flow, Lepidoptera, mimicry, molecular phylogeny, radiation, speciation, species delimitation

## Abstract

The evolutionary dynamics of diurnal *Aletis* moths (Lepidoptera: Geometridae: Sterrhinae) in the Afrotropics have been obscured by their allopatric distributions, significant inter‐ and intraspecific variation in adult and caterpillar phenotypes, and involvement in complex mimicry systems. Extensive phenotypic disparity, alongside conserved genital morphology and a lack of suitable material for genomic studies, has complicated species delineation. To elucidate species boundaries within *Aletis*, as well as explore their evolutionary history, divergence times, and patterns of population genetic structure, we collected fresh specimens of both caterpillars and adults across the *Aletis* distribution in South Africa and Uganda. We then conducted analyses using mitochondrial DNA and genome‐wide SNP data from ddRAD sequencing. Our finding supports the hypothesis of five distinct species in the study areas: *Aletis variabilis*, *A. helcita*, *A. erici*, *A. libyssa* and *A. concolor* . The mtDNA divergences range from 4.2% to 11.5%, while genomic data indicating diversification began 0.9 million years ago (Mya), and more recent divergence events occurred between 0.35 and 0.27 Mya. In eastern South Africa, we identified distinct northern and southern genetic lineages, potentially shaped by Pleistocene isolation influenced by climate, whereas in Uganda, habitat and/or altitudinal variation appears to play a key role in their isolation. Notable genetic admixture was found within both northern and southern South African regions, along with gene flow from Uganda to northern South Africa, and extensive internal gene flow among southern populations. We conclude that habitat fragmentation, leading to the patchy occurrence of caterpillar host plants, has contributed to increased genetic isolation and allopatric speciation. We also emphasize the critical conservation needs for preserving genetic diversity, which is essential for resilience in the rapidly changing Afrotropical landscape.

## Graphical abstract

We investigated the evolutionary history and species boundaries of diurnal *Aletis* moths in the Afrotropics using mitochondrial DNA and genome‐wide SNP data. Our results support five distinct species, with divergence dating back to 0.9 million years ago, influenced by climate‐driven isolation and habitat variation. These findings highlight the role of habitat fragmentation in shaping genetic diversity and emphasize the need for conservation efforts.

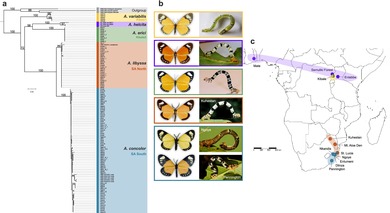

## 1 Introduction

The Afrotropical region, encompassing sub‐Saharan Africa, Madagascar, and adjacent Islands, is recognized for its extraordinary biodiversity and high levels of endemism, making it a hotspot for evolutionary research and a quagmire for taxonomic studies (Raven et al. 2020; Williams 2024). The geometrid moths are a prime example of this phenomenon, with c. 4000 of the c. 24,000 species described globally occurring the Afrotropical region (Rajaei et al. 2022; Scoble 1999). Like in other parts of the world, most of the African geometrid fauna was described in the late 19th and early 20th centuries (Gaston et al. 1995), during a period when taxonomic work primarily relied on a limited morphological character. Recent taxonomic work on the African geometrids has mostly focused on the description of single species, revisions of species groups or genera, and molecular phylogenies that place African taxa within a broader phylogeographical context (see Rajaei et al. 2022). Several recent studies have incorporated not only museum specimens and morphological analysis, but also molecular data, often using DNA barcodes, and life history traits to strengthen taxonomic conclusions (e.g., Staude et al. 2020 and references therein). However, in some complex cases, these data may still be insufficient to resolve intricate taxonomic relationships or delineate species boundaries, necessitating an integrative approach that includes genome‐level data (e.g., Padial et al. 2010). Such an approach is particularly relevant to the Afrotropical genus *Aletis*, whose complex taxonomy and evolutionary history remain only partially understood.

*Aletis* Hübner, 1820 are diurnal geometrid moths endemic to the Afrotropics, notable for their butterfly‐like appearance, which has contributed to a complex taxonomic history. The type species, *A. helcita* (Linnaeus, 1763), was originally classified in the butterfly genus *Papilio* (*Danaus*) (Linnaeus, 1763) but was later reassigned to the Geometridae. While *Aletis* has historically been placed in the subfamily Oenochrominae (Prout 1929–35, Janse 1933–35), recent morphological and molecular phylogenies have strongly supported its classification within Sterrhinae: Scopulini (Holloway 1997; Sihvonen 2005; Sihvonen et al. 2020). In Sihvonen's (2005) revision, *Aletis* and *Cartaletis* were temporarily treated as synonyms of *Scopula*, reflecting their close relationship within Scopulini. However, subsequent integrative analyses reinstated *Aletis* as a valid genus and recognized *Cartaletis* Warren, 1894, as its junior synonym (Sihvonen et al. 2020). There are 39 nomenclaturally available names across these two genera, classified into 12 species and 12 subspecies, with 15 synonyms in total (Rajaei et al. 2022; Scoble 1999). In South Africa, three species have been recorded (Krüger 2020).

**FIGURE 1:**
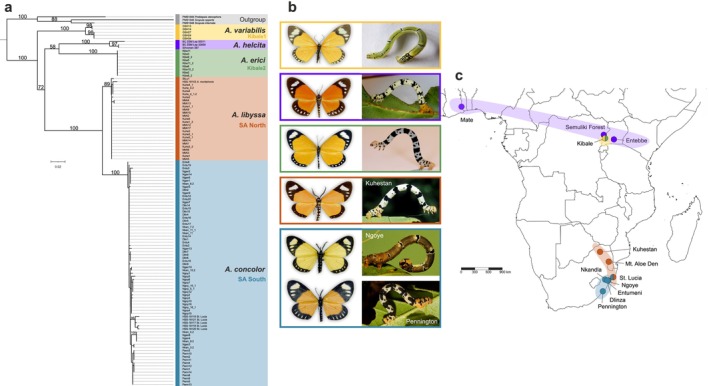
Maximum‐likelihood tree based on the mtDNA dataset, with representative habitus images of select lineages. (a) The ML tree is rooted by three outgroups: *Scopula internata*, *S. opperta*, and *Problepsis ctenophora*. Branch numbers indicate ML bootstrap support values. (b) *Aletis* adults and caterpillars, each with variable patterns, display aposematic signals that advertise their poisonous or distasteful properties with warning colouration. Their diurnal lifestyle and butterfly‐like appearance have contributed to a complex taxonomic history concerning species boundaries and phylogenetic placement. (c) Geographical distribution of the sampled *Aletis* species, with population labels on the map. Colors correspond to lineages identified in phylogenetic analyses (a).

*Aletis* is part of a mimicry complex involving other Lepidoptera, including *Aletopus dargei* (Noctuidae), *Phaegorista similis* (Erebidae), *Mesomima tmetoleuca, Mimaletis watulekii*, and several species of *Zerenopsis* (Geometridae), as well as *Hypolimnas misippus* , *Euphaedra ruspina*, and *Danaus chrysippus* (Nymphalidae) (Staude and Curle 1997; Staude and Sihvonen 2014; Sihvonen et al. 2021). Even behavioral mimicry has been observed by the authors of the present paper in South Africa, where the sympatric *Telchinia esebria* (Nymphalidae) and *Aletis concolor* fly in a similar fluttering manner and elevation. When disturbed, *Telchinia esebria* starts to fly in a powerful, zigzag motion typical of butterflies (unpublished observation). Furthermore, assumed *Aletis* species are uniform within populations but display slight variations in wing colouration and caterpillar patterns across allopatric populations (see Figure 1b). The genitalia and similar between assumed species, making morphology‐based identification and classification challenging, as shown by the excessive synonymy (Scoble 1999).

*Aletis* moths feed on various *Oxyanthus* spp. (Rubiaceae), also an endemic forest genus native to tropical and southern Africa, occurring in the remaining forest patches. These plants contain several toxic bioactive compounds, including cycloheptyl cyanide and cyanogenic glycosides, common defensive traits in species of Apocynaceae and Asclepiadaceae (Ebigwai et al. 2020; Rockenbach et al. 1992). Despite these chemical defenses, *Aletis* caterpillars have adapted to exploit the leaves of this plant as a primary resource, although they have also been recorded feeding on other Rubiaceae species, such as *Randia armata*, which may possibly be due to misidentification (Robinson et al. 2023; Staude 1999; Staude et al. 2020). The current patchy distribution of *Aletis* in South Africa mirrors that of *Oxyanthus*. Evidence from pollen samples suggests that forest fragmentation occurred several times during the Pleistocene and Holocene (Neumann et al. 2024), leading to the formation of allopatric populations and increased genetic isolation.

Based on extensive sampling from eastern South Africa and other locations within the range, we provide genomic insights into species delimitation and the evolutionary dynamics of *Aletis* populations and species. Using both mitochondrial DNA and genome‐wide SNP data from ddRAD sequencing, we investigate population structure, divergence timing, and species boundaries, focusing on genetic divergence shaped by both historical and modern processes. This comprehensive genomic analysis offers new insights into the drivers of divergence, adaptation, and biodiversity in the Afrotropics.

## 2 Material and Methods

### 2.1 Taxon Sampling and Preliminary Identification

We initially collected 111 specimens of *Aletis* from Uganda and South Africa, including three outgroup species belonging to Sterrhinae: Scopulini (*Problepsis ctenophora*, *Scopula opperta*, and *Scopula internata*), which served for rooting the phylogenetic trees. Among the samples analyzed, 17% were adult leg tissues and 83% were caterpillars, all prepared for molecular analyses (see Table S1). In South Africa, molecular analyses were conducted on 94 individuals, including both caterpillars and adults. Most caterpillars were collected directly from the field from multiple host plants, and a few were reared to adulthood to confirm adult morphology. Samples were obtained from eight allopatric localities in January 2023. Field samples were labeled, and 2–3 legs from each adult individual and the entire body of each caterpillar, were immediately preserved in 96% ethanol for subsequent molecular analysis. Ugandan samples, also including both caterpillars and laboratory reared adults, were collected from Kibale National Park in November 2019 and were treated similarly. Within Kibale, individuals were sampled from both the northern sector (evergreen moist forest, 1300–1400 m) and the southern sector (lower‐altitude forest, 1100–1200 m).

Field‐collected specimens were given preliminary identifications, with collecting site specific codes, based on the literature (Prout 1929–1935, Janse 1933–1935, Krüger 2020) and comparison of adults against the type specimens in the following museums: The Natural History Museum London (United Kingdom), Natural History Museum Berlin (Germany), Bavarian State Collection of Zoology Berlin (Germany) and Ditsong National Museum of Natural History Pretoria (South Africa).

### 2.2 DNA Extraction and mtDNA Sequencing

Genomic DNA (gDNA) was extracted from a couple of adult legs, or from whole caterpillar bodies for smaller specimens (up to 1.5 mg) and partial bodies for larger ones (up to 3 mg) using the DNeasy Blood and Tissue kit (Qiagen), following the manufacturer's protocol. The gDNA concentration was checked using the Quant‐iT PicoGreen dsDNA assay kit (Molecular Probes). A partial 648 bp fragment of the mitochondrial COI gene (mtDNA), widely used for DNA barcoding, was amplified following the primers and protocol proposed by Wahlberg and Wheat (2008). PCR products were purified using ExoSAP‐IT (Thermo Fisher Scientific), and sanger sequenced in the forward direction at FIMM (Helsinki, Finland). We downloaded nine published *Aletis* sequences from GenBank and BOLD Systems (Ratnasingham and Hebert 2007), incorporating them into the newly sequenced dataset. The final sequence dataset was aligned using MEGA (Tamura et al. 2021), and pairwise distances were calculated with the Kimura 2‐paramter (K2P) model. The newly produced sequences were assigned GenBank accession numbers (see Table S1).

### 2.3 ddRAD‐seq Library Preparation and Sequencing

A total of 96 samples including three outgroup species was selected from the mtDNA dataset, representing distinct lineages. *Aletis helcita* samples were excluded due to the lack of fresh material. The ddRAD‐seq library preparation was performed following the protocols of Lee et al. (2018), with some modifications as follows: the gDNA was digested using PstI and MseI restriction enzymes (both from NEB). Post digestion, fragments were manually purified with AMPure XP magnetic beads (Agencourt). The DNA fragments were size selected at 300 bp using the Pippin Prep (Sage Science), and the size distribution was assessed using a Bioanalyzer (Agilent Technologies). The final library was sequenced on an Illumina NovaSeq X Plus PE150 at Novogene (Munich, Germany).

### 2.4 ddRAD‐seq Bioinformatics

Raw paired‐end reads were demultiplexed using their unique index and adapter sequences, with no mismatches tolerated, using *ipyrad* v.0.9.92 (Eaton and Overcast 2020). The demultiplexed paired reads were processed with PEAR v.0.9.8 (Zhang et al. 2014) using default setting to merge overlapping reads, and then input into the *ipyrad* pipeline for further processing. Initial filtering steps, single nucleotide polymorphism (SNP) calling, and sequence alignment were carried out. Several parameters were adjusted from the default settings after testing various combinations: datatype was set to “ddrad”, assembly method to “denovo,” restriction overhang to “TGCAG,TAA,” maximum low‐quality bases to 6, minimum statistical depth to 8, clustering threshold to 0.9, and minimum number of samples with a given locus to 20. We generated two types of final matrices: one including all variable sites (SNPs) and another containing one random SNP from each putatively unlinked locus (uSNPs). These matrices were designed for different analyses, depending on the data volume, which could affect the speed of analyses or be limited by the large dataset size.

### 2.5 Mitochondrial Gene Tree and Genetic Clustering

Maximum likelihood (ML) inference was conducted using IQ‐TREE v.2.1.3 (Minh et al. 2020), where the best‐fitting substitution model was selected via ModelFinder (Kalyaanamoorthy et al. 2017). Node support was assessed with ultrafast bootstrap approximations (Hoang et al. 2018). The resulting trees were visualized in FigTree v.1.4.2 (Rambaut 2015). The resulting figures were modified as needed using CorelDRAW v24 and Adobe Illustrator CS6.

### 2.6 Population Genomic Structure

We used the unlinked SNPs (uSNPs) dataset to study population genomic structure, examining the number and geographic distribution of genetically distinct groups. First, we ran STRUCTURE v.2.3.4 (Pritchard et al. 2000) to detect potential admixture among population clusters. Ten replicates were run for each *K* value, ranging from 1 and 7, with 20 K burn‐in generations and 100 K post burn‐in generations. StrAuto was used to automate Structure processing (Chhatre and Emerson 2017), and replicates were permuted using CLUMPP (Jakobsson and Rosenberg 2007). The optimal *K* was inferred using StructureHarvester (Earl and VonHoldt 2012), based on the ad hoc ∆*K* statistics (Evanno et al. 2005). In addition, we also analyzed subsets of South African populations with the same parameters as above to investigate fine‐scale structure. STRUCTURE results were visualized using Distruct (Rosenberg 2004).

We next employed SplitsTree v.4.19.2 using the Neighbor‐Net algorithm with uncorrected p‐distances to generate an unrooted genetic network (Huson and Bryant 2006). Pairwise *F*_st_ values were calculated using Arlequin v.3.5.1.3 (Excoffier and Lischer 2010) with 1000 permutations.

### 2.7 SNP Phylogenies and Coalescence Species Trees

A phylogenomic ML tree was constructed using the SNP dataset in IQ‐TREE and rooted to Kibale 1 and 2. We used the “Auto” option for best‐fit substitution model and tested nodal support with 1000 bootstrap replicates. We also applied a coalescent approach using the SNAPPER module (Stoltz et al. 2021) in BEAST v.2.6.6 (Bouckaert et al. 2019). Two independent runs of SNAPPER were conducted with default settings for five million generations. Convergence (ESS > 200) was checked using Tracer v.1.7.2 (Rambaut et al. 2018), and burn‐in was set to 20%. The posterior distribution of trees was evaluated with DensiTree v.2.2.1 (Bouckaert 2010), and a maximum clade credibility (MCC) tree was generated using TreeAnnotator.

### 2.8 Species Delimitation

To delineate possible species boundaries within *Aletis* species, we used the SPEEDEMON v.1.1.0 package (Douglas and Bouckaert 2022) in BEAST v.2.7.1 analyzing 93 individuals excluding three outgroups. The unlinked SNPs (uSNPs) dataset served as input, and each population was hypothesized as an independent species for species delimitation under the multi‐species coalescent model. Following the developer's guidance (https://github.com/rbouckaert/speedemon), we employed the Yule Skyline Collapse model (Duran et al. 2024), where samples with an estimated ancestral species time below the epsilon cut‐off of 10^−4^ were collapsed into a single species. We conducted two million MCMC generations, leaving all other model parameters at their default settings. We assessed the convergence (ESS > 200) using Tracer, and the multi‐species model was analyzed with the ClusterTreeSetAnalyser to evaluate whether distinct populations could be merged into a single species based on the 93 individuals.

Additionally, we performed Bayes factor species delimitation (BFD*) (Leaché et al. 2014), implemented via the SNAPP (Bryant et al. 2012) and Path Sampler packages in BEAST v.2.6.6 to validate and compare the results with those from SPEEDEMON, providing support for the robustness of our findings. The uSNPs dataset was also used as input for this analysis. To estimate the marginal likelihood of each species delimitation model, we conducted a stepping‐stone analysis (*α* = 0.3) using 12 steps, with an MCMC length of 250 K generations and a 25 K pre‐burn‐in, followed by a 10% final burn‐in. Sampling frequency was evaluated, and convergence (ESS > 200) was checked using Tracer. The species delimitation models were ranked by their marginal likelihoods, and Bayes Factors were calculated to compare the models (Kass and Raftery 1995).

### 2.9 Estimation of Migration Events

To investigate the admixture history within *Aletis* species, we applied the population tree inference model implemented in TreeMix v.1.13 (Pickrell and Pritchard 2012). The analysis was performed on the uSNPs dataset. Individuals were assigned to populations, and migration events were progressively added up to a total of 8. Asymmetries in the covariance matrix of allele frequencies, relative to the ancestral population as inferred from the maximum likelihood tree, were used to identify putative gene flow among populations. TreeMix infers historical admixture signals rather than contemporary migration, providing insights into past admixture events that have shaped the genetic structure of populations. However, the direction and magnitude of inferred migration edges should be interpreted cautiously, as these results are considered exploratory rather than definitive (see https://speciationgenomics.github.io/Treemix/).

## 3 Results

### 3.1 Divergence Patterns Inferred From mtDNA Data

Maximum likelihood analysis of the mtDNA dataset (120 sequences of 648 bp) reliably differentiated five distinct lineages within the genus *Aletis*: *A. variabilis* , *A. helcita*, *A. erici*, *A. libyssa*, and *A. concolor* (Figure 1a). In Kibale National Park, Uganda, two lineages, *A. variabilis* and *A. erici*, were identified (Figure 1c), while *A. helcita* exhibited a wide geographical range, extending from Ghana to Uganda. In South Africa, *A. libyssa* and *A. concolor* were confined to distinct regions, with *A. libyssa* occurring in northern areas and *A. concolor* in southern regions. In St. Lucia, six individuals were assigned to two species, *A. libyssa* and *A. concolor* . Only one *A. libyssa* individual from this population could be included in the ddRAD‐seq analyses, as fresh material of *A. concolor* from this locality was unavailable. The mtDNA divergence revealed deep pairwise interspecific variation, ranging from 4.2% to 11.5% (Table 1). The lowest divergence was observed between *A. libyssa* and *A. concolor* at 4.2%, while the highest divergence of 11% was observed between *A. variabilis* and *A. erici*, despite their co‐occurring in Kibale National Park.

**TABLE 1:** Estimated pairwise *F*_ST_ based on SNP data (below diagonal) and mean *p*‐distances based on K2P for mtDNA data (above diagonal).

Group	Population	Kiba1	Kiba2	Kuhe	Mt.Al	St.Lu	Nkan	Ngoy	Dlin	Entu	Penn
Kibale1	Kibale1	—	10.9	9.9	9.9	10.0	11.4	11.5	11.2	11.2	11.1
Kibale2	Kibale2	0.877*	—	8.9	8.8	8.8	10.6	10.6	10.6	10.6	10.2
SA North	Kuhestan	0.913*	0.928*	—	0.1	0.4	3.7	4.0	3.7	3.6	3.6
	Mt. Aloe Den	0.877*	0.896*	0.244*	—	0.4	3.8	4.0	3.7	3.6	3.7
	St. Lucia	0.910	0.950	0.522	0.213	—	3.6	3.9	3.6	3.6	3.7
SA South	Nkandla	0.970*	0.975*	0.877*	0.820*	0.964	—	0.6	0.4	0.3	0.6
	Ngoye	0.955*	0.964*	0.848*	0.782*	0.924	0.381*	—	0.5	0.5	0.5
	Dlinza	0.966*	0.972*	0.868*	0.809*	0.950	0.110*	0.274*	—	0.1	0.5
	Entumeni	0.968*	0.974*	0.877*	0.822*	0.957	0.144*	0.280*	0.006*	—	0.5
	Pennington	0.949*	0.958*	0.836*	0.767*	0.899	0.450*	0.375*	0.393*	0.434*	—

*Note:* Significant *p*‐values (< 0.05) for *F*_ST_ are indicated with an asterisk.

### 3.2 Genome‐Wide SNP Data

Illumina sequencing of the ddRAD library initially included 96 individuals, along with three outgroups species. However, one sample (GSH14) was excluded due to low quality reads. For the remaining 92 samples excluding three outgroups, the mean number of reads per individual was approximately 1 million (Table S2). After assembly and retaining loci shared across at least 20 individuals, the average number of loci was 7099. The final sequence matrix consisted of 2,784,431 bp, including 135,834 SNPs and 80,826 parsimonious informative sites, accommodating up to 43% missing data.

### 3.3 Genetic Clusters

Population clustering analyses using STRUCTURE identified six distinct genetic groups and revealed notable admixture among populations (Figure S1). Consistent with the ML tree, two distinct clusters were observed in Kibale National Park.

In South Africa, two geographically distinct genetic clusters were identified, corresponding to the northern (red) and southern (blue) regions (Figure S2b). Further analysis of northern South African populations revealed two groups: Kuhestan and a combined group of Mt. Aloe Den + St. Lucia. Individuals from the Mt. Aloe Den + St. Lucia group showed a predominant assignment (ca. 40%, orange) to a cluster specific to these populations, while also sharing a substantial proportion (ca. 60%, red) with Kuhestan. In southern South Africa, populations also split into two primary clusters: Entumeni + Nkandla + Dlinza + Ngoye, and Pennington. Individuals from Pennington exhibited a predominant assignment (ca. 65%, purple) to a cluster largely specific to this population, while sharing a proportion of ancestry (ca. 35%, blue) with the common southern cluster present in the other southern populations. Because sampling intensity varied among populations, we interpret STRUCTURE's optimal K (which captures both inter‐ and intraspecific structure) with caution and in the context of concordant TreeMix results.

Both SplitsTree networks showed similar clustering patterns but failed to detect sub‐clusters within northern and southern South African populations (Figure S2a). Genetic differentiation between clusters was further supported by pairwise *F*_ST_ values (Table 1).

### 3.4 Phylogenomics and Estimation of Divergence Times

**FIGURE 2:**
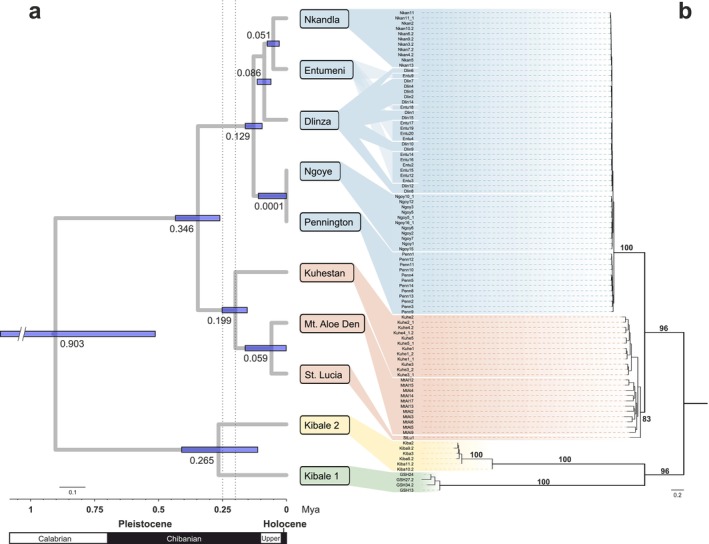
(a) Divergence times estimated from SNAPP for 10 populations. Node bars represent the 95% highest posterior density intervals for estimated divergence times for each node. The mean age estimate for each node is given near the bar. (b) Phylogenomic results based on IQ‐TREE analysis of ddRAD SNP data. Bootstrap values are indicated near the branch of the tree. Colors correspond to lineages identified in phylogenetic analyses using mtDNA (see Figure 1).

**FIGURE 3:**
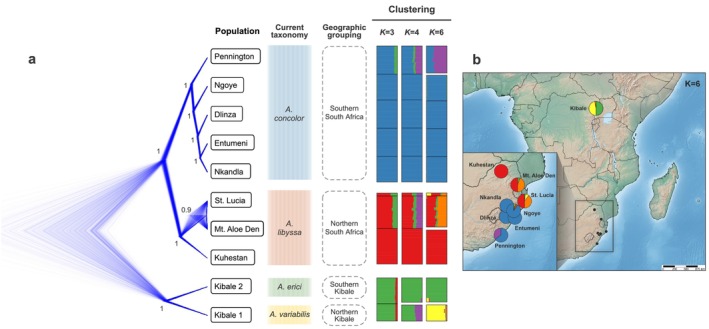
(a) Species tree obtained for 10 populations shown as a cloudogram from DensiTree. Posterior probabilities are presented for the relevant clades. Genetic clusters according to STRUCTURE analysis at *K* = 3, 4, and 6. Each bar represents an independent lineage supported by the analysis. (b) Pie chart map following *K* = 6 results, showing population‐genomic admixture according to geographic location.

Our phylogenomic hypothesis, based on ddRAD SNP data, robustly supported the presence of four distinct lineages: Kibale 1, Kibale2, northern South Africa, and southern South Africa, corresponding to lineages identified in the mtDNA phylogeny (Figure 2b). Each lineage was supported with maximum bootstrap values, except for the northern South African lineage (comprising Kuhestan, Mt. Aloe Den, and St. Lucia), which had a bootstrap support of 83%. The species tree analysis was consistent with the ML tree (Figure 3).

Divergence time estimates indicated that the genus *Aletis* began to diversify during the early Calabrian stage of the Pleistocene, ca. 0.9 Mya (Figure 2a). The Kibale1 and Kibale2 lineages diverged ca. 0.26 Mya, while the South African lineages started to diverge at ca. 0.35 Mya during the middle of the Chibanian stage. The northern South African lineage diverged from the southern populations around 0.2 Mya, with the southern lineage representing the most recent divergence at 0.13 Mya.

### 3.5 Species Boundaries Within *Aletis*

Species delimitation analyses were conducted using a multi‐species coalescent approach with predefined population assignments: Kibale1, Kibale2, Kuhestan, Mt.Aloe Den, St. Lucia, Nkandla, Entumeni, Dlinza, Ngoye, and Pennington. Of the 10 competing species hypotheses, both BFD* (see Table S3) and SPEEDEMON (88.4% posterior support) consistently supported the four‐species model: Kibale1 ( *A. variabilis* ), Kibale2 (*A. erici*), the northern South African populations (grouping Kuhestan, Mt. Aloe Den, and St. Lucia as *A. libyssa*), and the southern South African populations (grouping Nkandla, Entumeni, Dlinza, Ngoye, and Pennington as *A. concolor* ) as the most likely species boundaries. The molecular data therefore supports the view that the observed variation in caterpillar color and pattern, as well as variation in wing hues among southern South African *Aletis concolor* populations represent intraspecific variation (see Figure 1b).

### 3.6 Gene Flow

**FIGURE 4:**
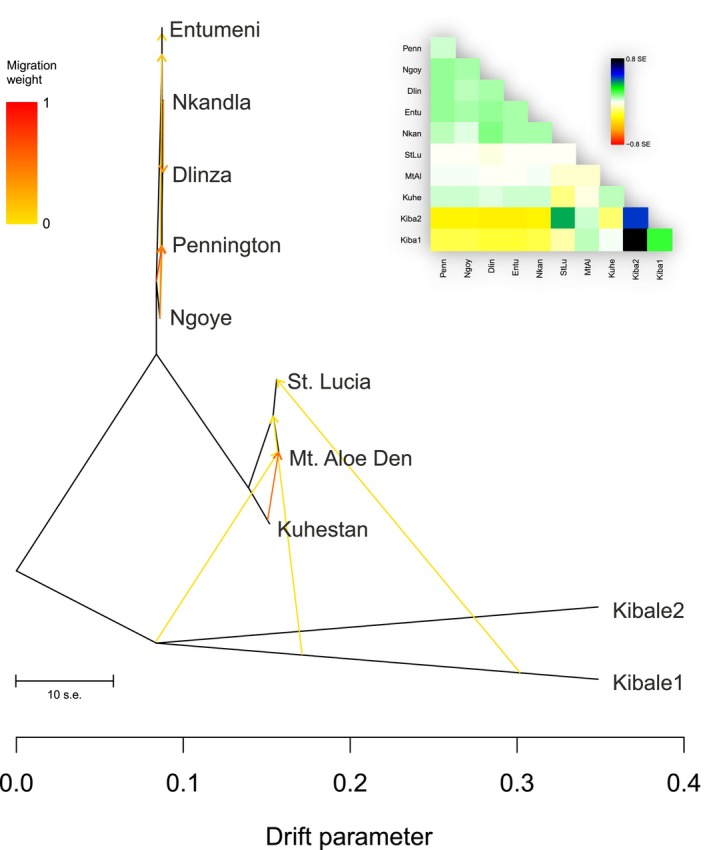
TreeMix results based on unlinked SNP data. Branch lengths are proportional to the evolutionary change (the drift parameter), and terminal nodes were labeled with populations. The scale bar represents 10 times the average standard error (SE) of the values in the covariance matrix, the migration weight represents the fraction of ancestry derived from the migration edge. The residual covariance between each pair of populations is shown in the upper right corner. Residuals above zero indicate populations that are more closely related to each other in the data than in the best‐fit model, suggesting possible admixture events.

To further validate the suggested admixture within *Aletis*, we computed an allele frequency‐derived ML tree using TreeMix, incorporating potential admixture events. Kibale1 and Kibale2, as sister lineages, were positioned at the base of the unrooted tree (Figure 4). TreeMix identified several putative migration events: three migration events from Kibale1 to the northern South African populations, and one internal gene flow event from Kuhestan to Mt. Aloe Den within northern South African populations. Interestingly, no external gene flow was detected into the southern South African populations, while multiple internal signals were observed among them, indicating relative genetic isolation from other populations.

## 4 Discussion

Both mtDNA and genomic SNP data revealed strong congruence, confirming the presence of five distinct *Aletis* lineages in the examined populations within the Afrotropical region: *A. variabilis* , *A. helcita*, *A. erici*, *A. libyssa*, and *A. concolor* . The genetic differentiation observed between *A. variabilis* and *A. erici* in Kibale National Park, Uganda, coincides with ecological differences, particularly habitat and altitudinal isolation variation, suggesting these factors may contribute to their divergence. Notably, despite extensive sampling of caterpillars in the nothern part of Kibale National Park, which represents evergreen moist forest at altitudes of approximately 1300–1400 m between 2011 and 2021 (unpublished data by Sile Holm), *A. erici* has not been collected in this region. Instead, it is confined to the savannah‐influenced southern part of the park at lower altitudes around 1100–1200 m. This ecological partitioning, together with the estimated divergence time of 265,000 years ago, points to a possible role of ecological factors in shaping differentiation. Other processes, such as historical range dynamics, spatial isolation, or potential host‐plant associations (which were not identified in this study), may also have contributed to divergence. The apparent wide distribution of *A. helcita*, spanning from Ghana to Uganda based on three individuals, suggests potential adaptability and ecological plasticity, consistent with observations in other Lepidoptera that demonstrate resilience to climatic fluctuations (Valtonen et al. 2013); however, additional sampling is needed to confirm these patterns.

In South Africa, the divergence of the northern (= *A. libyssa*) and southern (= *A. concolor* ) lineages around 346,000 years ago, likely reflects Pleistocene climate‐driven refugial isolation. During this period, Africa experienced significant environmental shifts, with cooler and drier conditions leading to widespread habitat contraction (deMenocal 2004; Hewitt 2004). Northerly regions and coastal forests along the eastern seaboard likely remained habitable, serving as refugial zones (Lawes et al. 2007). Northern populations, which show higher genetic diversity and admixture, may reflect persistence in or recolonization from multiple refugial areas, whereas southern populations likely persisted in more localized coastal refugia. Similar patterns of refugial isolation and recolonization have been observed in other species during the Pleistocene glaciations (Spitzweg et al. 2019).

Modern, human‐induced forest fragmentation in South Africa, particularly along the KwaZulu‐Natal coastal strip, has likely contributed to genetic isolation in *Aletis* species. Over the past 150 years, human encroachment, including urbanization and agricultural activities like sugarcane cultivation, has led to deforestation (Naicker et al. 2016). In contrast, habitat fragmentation affecting southern *A. concolor* populations in the inland scarp forests around Eshowe (KwaZulu‐Natal) appears to have been primarily driven by long‐term climate change (Lawes et al. 2007). The intervening areas are dominated by grasslands and drier savanna habitats, which are unsuitable for forest growth due to significantly lower rainfall. Thus, *A. libyssa* (northern populations) likely persisted in coastal refugia, while *A. concolor* (southern populations) may have survived in humid forest patches of southern KwaZulu‐Natal.

The lack of detectable gene flow between these lineages, despite being approximately 150 km apart at the closest location, underscores the combined influence of long‐term refugial isolation and more recent human‐induced fragmentation on their genomic divergence and current distribution. Although TreeMix indicated possible historical admixture among lineages, the signals should be interpreted cautiously, as direction and timing cannot be fully resolved with the current data. This isolation is consistent with patterns of high endemicity in South Africa across a wide range of taxa, such as reptiles, plants, and freshwater crabs (Gouws et al. 2015; Hughes et al. 2005; Vamberger et al. 2018).

While our current dataset represents the best available material from Uganda and South Africa, we recognize that the wide unsampled corridor between them could include intermediate populations that influence patterns of divergence. Addressing this gap through targeted genomic and ecological sampling will be an important future step to assess whether the observed lineages are separated by sharp breaks or connected by gradual transitions.

*Aletis* moths exhibit considerable morphological variation, particularly in adult wing colouration (Janse 1933–1935) and caterpillar patterns, across localities, which may reflect population‐level divergence rather than individual plasticity. Such variation, possibly shaped by local environmental factors like vegetation type and microclimates, contrasts with the lack of genital differences across species. The discrepancy between the morphological traits and the clear genetic differentiation highlights that traditional morphological criteria alone may not fully capture species boundaries. Integrative approaches that combine genetic, ecological, and morphological data are essential for accurately discerning speciation and evolutionary patterns in these moths, as well as other organisms.

Our findings highlight how historical climatic events and ecological factors, such as habitat fragmentation and altitude isolation, have shaped current biodiversity patterns in Afrotropical *Aletis* populations. Our sampling remains limited, particularly within the potential contact zone between *A. libyssa* and *A. concolor* (e.g., St. Lucia in KawaZulu‐Natal), where both species co‐occur. As only a single *A. libyssa* individual from this locality was included in the genomic dataset, the data cannot fully capture the genetic composition or species diversity of the local population. Consequently, it remains unresolved whether the observed incongruence between mtDNA and morphology identification in this population reflects non‐diagnostic traits or potential mitochondrial introgression. Beyond this locality, denser and more targeted sampling in KwaZulu‐Natal and Central Africa, where *A. helcita* is widespread, will be crucial to determine whether lineages meet in parapatry, maintain isolation, or show admixture. High‐performance clustering tools such as PopCluster (Bailey 2025; Wang 2024) could enhance inference of subtle population structure and admixture in future studies. Such efforts will further clarify the evolutionary status of highly differentiated populations such as Pennington within *A. concolor* . From a conservation perspective, the distinct genetic signatures of northern and southern *Aletis* populations in South Africa support the need for tailored conservation strategies to preserve unique genetic diversity. Protecting key habitats, especially refugial zones, can enhance species' resilience to ongoing environmental change (Eeley et al. 2001; Jenkins et al. 2013).

## Author Contributions

**Kyung Min Lee:** conceptualization (lead), data curation (lead), formal analysis (lead), investigation (lead), methodology (lead), project administration (lead), software (lead), validation (lead), visualization (lead), writing – original draft (lead), writing – review and editing (lead). **Hermann Staude:** conceptualization (equal), investigation (equal), resources (lead), validation (equal), writing – review and editing (supporting). **Sille Holm:** data curation (equal), investigation (equal), resources (equal), writing – review and editing (supporting). **Elina Laiho:** data curation (supporting), methodology (supporting), project administration (supporting), writing – review and editing (supporting). **Leidys Murillo‐Ramos:** resources (supporting), writing – review and editing (supporting). **Øystein H. Opedal:** resources (supporting), writing – review and editing (supporting). **Hossein Rajaei:** resources (supporting), writing – review and editing (supporting). **Reinier F. Terblanche:** resources (supporting), validation (supporting), writing – review and editing (supporting). **Magda Botha:** investigation (supporting), resources (supporting), writing – review and editing (supporting). **Pasi Sihvonen:** conceptualization (lead), data curation (equal), funding acquisition (lead), investigation (equal), project administration (lead), resources (lead), validation (lead), writing – original draft (lead), writing – review and editing (lead).

## Funding

This work was supported by the Pentti Tuomikoski Fund; Research Council of Finland, 331995.

## Conflicts of Interest

The authors declare no conflicts of interest.

## Supporting information


**Table S1:** Information on sample collection and mtDNA success for the studied taxa, including three outgroup species. Specimens were sourced from Luomus (Finnish Museum of Natural History, Finland), SNSB (SNSB Zoologische Staatssammlung, Germany), and NHM (Natural History Museum London, UK).
**Table S2:** ddRAD data summary.
**Table S3:** Different species delimitation models for the group evaluated with the BFD* method and their results. Each row indicates different species scenarios. The best scenario is shown in bold. Abbreviations refer to each population: kiba1, kiba2, SA_North (kuhe, MtAl, StLu), SA_South (Entu, Dlin, Nkan, Ngoy, Penn).
**Figure S1:** STRUCTURE plots generated using different values of estimated clusters (K = 2 to 7). This plot indicates ancestry proportions from K inferred genetic groups. Summary of mean LnP(K), standard deviation, and delta K values were calculated following Evanno et al. (2005).
**Figure S2:** SplitsTree built using uncorrelated P distances based on SNP data.

## Data Availability

The mitochondrial DNA sequences generated in this study are accessible via NCBI GenBank under the following accession numbers: PQ373211‐PQ373330 (as listed in Table S1). The ddRAD raw reads are deposited at NCBI SRA under BioProject no. PRJNA1172417 and BioSample accession numbers SAMN44271940‐ SAMN44272031.
