# Supplementary material for: Genomic Insights Into Species Delimitation and the Evolutionary History of Mimetic Aletis Moths (Lepidoptera, Geometridae) in the Afrotropics
Source: Ecol Evol. 2025 Dec 18;15(12):e72745. doi: 10.1002/ece3.72745 (PMC12714424; doi:10.1002/ece3.72745)
Supplement: Supplementary file 1 — Table S1: Information on sample collection and mtDNA success for the studied taxa, including three outgroup species. Specimens were sourced from Luomus (Finnish Museum of Natural History, Finland), SNSB (SNSB Zoologische Staatssammlung, Germany), and NHM (Natural History Museum London, UK). Table S2: ddRAD data summary. Table S3: Different species delimitation models for the group evaluated with the BFD* method and their results. Each row indicates different species scenarios. The best scenario is shown in bold. Abbreviations refer to each population: kiba1, kiba2, SA_North (kuhe, MtAl, StLu), SA_South (Entu, Dlin, Nkan, Ngoy, Penn). Figure S1: STRUCTURE plots generated using different values of estimated clusters (K = 2 to 7). This plot indicates ancestry proportions from K inferred genetic groups. Summary of mean LnP(K), standard deviation, and delta K values were calculated following Evanno et al. (2005). Figure S2: SplitsTree built using uncorrelated P distances based on SNP data. [file ECE3-15-e72745-s001.docx]

**Supporting information**

**Table S1.** Information on sample collection and mtDNA success for the studied taxa, including three outgroup species. Specimens were sourced from Luomus (Finnish Museum of Natural History, Finland), SNSB (SNSB Zoologische Staatssammlung, Germany), and NHM (Natural History Museum London, UK).

| Species | Population | Country | Sample ID | Tissue type | GenBank accession | Collection date | Lat/Lon | Resources |
| --- | --- | --- | --- | --- | --- | --- | --- | --- |
| *Problepsis ctenophora* | - | South Africa | PMS1545 | 2-3 legs | PQ373211 | N/A | N/A | Luomus |
| *Scopula opperta* | - | South Africa | PMS1546 | 2-3 legs | PQ373212 | N/A | N/A | Luomus |
| *Scopula internata* | - | South Africa | PMS1548 | 2-3 legs | PQ373213 | N/A | N/A | Luomus |
| *Aletis helcita* | Mate | Ghana | BC ZSM Lep 00011 | N/A | PQ373328 | N/A | 7.94, -1.22 | SNSB |
| *A. helcita* | Semuliki | Uganda | BC ZSM Lep 32830 | N/A | PQ373329 | N/A | 0.79,30.03 | SNSB |
| *A. helcita* | Entebbe | Uganda | Sihvonen_387 | N/A | PQ373330 | N/A | 0.66,32.45 | NHM |
| *A. variabilis* | Kibale 1 | Uganda | GSH13 | 2-3 legs | PQ373214 | Nov. 2019 | 0.56,30.36 | Luomus |
| *A. variabilis* | Kibale 1 | Uganda | GSH14 | 2-3 legs | PQ373215 | Nov. 2019 | 0.56,30.36 | Luomus |
| *A. variabilis* | Kibale 1 | Uganda | GSH24 | 2-3 legs | PQ373216 | Dec. 2019 | 0.56,30.36 | Luomus |
| *A. variabilis* | Kibale 1 | Uganda | GSH27 | 2-3 legs | PQ373217 | Nov. 2019 | 0.56,30.36 | Luomus |
| *A. variabilis* | Kibale 1 | Uganda | GSH34 | 2-3 legs | PQ373218 | Nov. 2019 | 0.56,30.36 | Luomus |
| *A. erici* | Kibale 2 | Uganda | Kiba2 | caterpillar | PQ373219 | Nov. 2019 | 0.36,30.36 | Luomus |
| *A. erici* | Kibale 2 | Uganda | Kiba3 | caterpillar | PQ373220 | Nov. 2019 | 0.36,30.36 | Luomus |
| *A. erici* | Kibale 2 | Uganda | Kiba5 | caterpillar | PQ373221 | Nov. 2019 | 0.36,30.36 | Luomus |
| *A. erici* | Kibale 2 | Uganda | Kiba6 | caterpillar | PQ373222 | Nov. 2019 | 0.36,30.36 | Luomus |
| *A. erici* | Kibale 2 | Uganda | Kiba8_2 | caterpillar | PQ373223 | Nov. 2019 | 0.36,30.36 | Luomus |
| *A. erici* | Kibale 2 | Uganda | Kiba9_2 | caterpillar | PQ373224 | Nov. 2019 | 0.36,30.36 | Luomus |
| *A. erici* | Kibale 2 | Uganda | Kiba10_2 | caterpillar | PQ373225 | Nov. 2019 | 0.36,30.36 | Luomus |
| *A. erici* | Kibale 2 | Uganda | Kiba11 | caterpillar | PQ373226 | Nov. 2019 | 0.36,30.36 | Luomus |
| *A. erici* | Kibale 2 | Uganda | Kiba11_2 | caterpillar | PQ373227 | Nov. 2019 | 0.36,30.36 | Luomus |
| *A. libyssa* | Haenertsburg | South Africa | HSS-18143 | N/A | PQ373327 | N/A | -23.85,29.99 | SNSB |
| *A. libyssa* | Kuhestan | South Africa | Kuhe1 | caterpillar | PQ373301 | Jan. 2023 | -23.86,29.99 | Luomus |
| *A. libyssa* | Kuhestan | South Africa | Kuhe1_1 | caterpillar | PQ373302 | Jan. 2023 | -23.86,29.99 | Luomus |
| *A. libyssa* | Kuhestan | South Africa | Kuhe1_2 | caterpillar | PQ373303 | Jan. 2023 | -23.86,29.99 | Luomus |
| *A. libyssa* | Kuhestan | South Africa | Kuhe2 | caterpillar | PQ373304 | Jan. 2023 | -23.86,29.99 | Luomus |
| *A. libyssa* | Kuhestan | South Africa | Kuhe3 | caterpillar | PQ373305 | Jan. 2023 | -23.86,29.99 | Luomus |
| *A. libyssa* | Kuhestan | South Africa | Kuhe3_1 | caterpillar | PQ373306 | Jan. 2023 | -23.86,29.99 | Luomus |
| *A. libyssa* | Kuhestan | South Africa | Kuhe3_2 | caterpillar | PQ373307 | Jan. 2023 | -23.86,29.99 | Luomus |
| *A. libyssa* | Kuhestan | South Africa | Kuhe_4.2 | caterpillar | PQ373308 | Jan. 2023 | -23.86,29.99 | Luomus |
| *A. libyssa* | Kuhestan | South Africa | Kuhe4 | caterpillar | PQ373309 | Jan. 2023 | -23.86,29.99 | Luomus |
| *A. libyssa* | Kuhestan | South Africa | Kuhe4_1 | caterpillar | PQ373310 | Jan. 2023 | -23.86,29.99 | Luomus |
| *A. libyssa* | Kuhestan | South Africa | Kuhe4_1.2 | caterpillar | PQ373311 | Jan. 2023 | -23.86,29.99 | Luomus |
| *A. libyssa* | Kuhestan | South Africa | Kuhe5 | caterpillar | PQ373312 | Jan. 2023 | -23.86,29.99 | Luomus |
| *A. libyssa* | Kuhestan | South Africa | Kuhe5_1 | caterpillar | PQ373313 | Jan. 2023 | -23.86,29.99 | Luomus |
| *A. libyssa* | Mt. Aloe Den | South Africa | MtAl1 | caterpillar | PQ373314 | Jan. 2023 | -25.94,31.11 | Luomus |
| *A. libyssa* | Mt. Aloe Den | South Africa | MtAl2 | caterpillar | PQ373315 | Jan. 2023 | -25.94,31.11 | Luomus |
| *A. libyssa* | Mt. Aloe Den | South Africa | MtAl3 | caterpillar | PQ373316 | Jan. 2023 | -25.94,31.11 | Luomus |
| *A. libyssa* | Mt. Aloe Den | South Africa | MtAl4 | caterpillar | PQ373317 | Jan. 2023 | -25.94,31.11 | Luomus |
| *A. libyssa* | Mt. Aloe Den | South Africa | MtAl5 | caterpillar | PQ373318 | Jan. 2023 | -25.94,31.11 | Luomus |
| *A. libyssa* | Mt. Aloe Den | South Africa | MtAl6 | caterpillar | PQ373319 | Jan. 2023 | -25.94,31.11 | Luomus |
| *A. libyssa* | Mt. Aloe Den | South Africa | MtAl9 | caterpillar | PQ373320 | Jan. 2023 | -25.94,31.11 | Luomus |
| *A. libyssa* | Mt. Aloe Den | South Africa | MtAl12 | caterpillar | PQ373321 | Jan. 2023 | -25.94,31.11 | Luomus |
| *A. libyssa* | Mt. Aloe Den | South Africa | MtAl13 | caterpillar | PQ373322 | Jan. 2023 | -25.94,31.11 | Luomus |
| *A. libyssa* | Mt. Aloe Den | South Africa | MtAl14 | caterpillar | PQ373323 | Jan. 2023 | -25.94,31.11 | Luomus |
| *A. libyssa* | Mt. Aloe Den | South Africa | MtAl15 | caterpillar | PQ373324 | Jan. 2023 | -25.94,31.11 | Luomus |
| *A. libyssa* | Mt. Aloe Den | South Africa | MtAl17 | caterpillar | PQ373325 | Jan. 2023 | -25.94,31.11 | Luomus |
| *A. libyssa* | St. Lucia | South Africa | StLu1 | 2-3 legs | PQ373326 | Jan. 2023 | -28.37,32.42 | Luomus |
| *A. concolor* | St. Lucia | South Africa | HSS-18117 | N/A | PQ373296 | N/A | -28.37,32.42 | SNSB |
| *A. concolor* | St. Lucia | South Africa | HSS-18118 | N/A | PQ373297 | N/A | -28.37,32.42 | SNSB |
| *A. concolor* | St. Lucia | South Africa | HSS-18119 | N/A | PQ373298 | N/A | -28.37,32.42 | SNSB |
| *A. concolor* | St. Lucia | South Africa | HSS-18120 | N/A | PQ373299 | N/A | -28.37,32.42 | SNSB |
| *A. concolor* | St. Lucia | South Africa | HSS-18121 | N/A | PQ373300 | N/A | -28.37,32.42 | SNSB |
| *A. concolor* | Dlinza | South Africa | Dlin1 | caterpillar | PQ373228 | Jan. 2023 | -28.89,31.44 | Luomus |
| *A. concolor* | Dlinza | South Africa | Dlin2 | caterpillar | PQ373229 | Jan. 2023 | -28.89,31.44 | Luomus |
| *A. concolor* | Dlinza | South Africa | Dlin4 | caterpillar | PQ373230 | Jan. 2023 | -28.89,31.44 | Luomus |
| *A. concolor* | Dlinza | South Africa | Dlin5 | caterpillar | PQ373231 | Jan. 2023 | -28.89,31.44 | Luomus |
| *A. concolor* | Dlinza | South Africa | Dlin6 | caterpillar | PQ373232 | Jan. 2023 | -28.89,31.44 | Luomus |
| *A. concolor* | Dlinza | South Africa | Dlin7 | caterpillar | PQ373233 | Jan. 2023 | -28.89,31.44 | Luomus |
| *A. concolor* | Dlinza | South Africa | Dlin8 | caterpillar | PQ373234 | Jan. 2023 | -28.89,31.44 | Luomus |
| *A. concolor* | Dlinza | South Africa | Dlin9 | caterpillar | PQ373235 | Jan. 2023 | -28.89,31.44 | Luomus |
| *A. concolor* | Dlinza | South Africa | Dlin14 | caterpillar | PQ373236 | Jan. 2023 | -28.89,31.44 | Luomus |
| *A. concolor* | Dlinza | South Africa | Dlin15 | caterpillar | PQ373237 | Jan. 2023 | -28.89,31.44 | Luomus |
| *A. concolor* | Entumeni | South Africa | Entu2 | 2-3 legs | PQ373238 | Jan. 2023 | -28.88,31.46 | Luomus |
| *A. concolor* | Entumeni | South Africa | Entu3 | 2-3 legs | PQ373239 | Jan. 2023 | -28.88,31.46 | Luomus |
| *A. concolor* | Entumeni | South Africa | Entu4 | 2-3 legs | PQ373240 | Jan. 2023 | -28.88,31.46 | Luomus |
| *A. concolor* | Entumeni | South Africa | Entu9 | caterpillar | PQ373241 | Jan. 2023 | -28.88,31.46 | Luomus |
| *A. concolor* | Entumeni | South Africa | Entu12 | caterpillar | PQ373242 | Jan. 2023 | -28.88,31.46 | Luomus |
| *A. concolor* | Entumeni | South Africa | Entu14 | 2-3 legs | PQ373243 | Jan. 2023 | -28.88,31.46 | Luomus |
| *A. concolor* | Entumeni | South Africa | Entu15 | 2-3 legs | PQ373244 | Jan. 2023 | -28.88,31.46 | Luomus |
| *A. concolor* | Entumeni | South Africa | Entu16 | 2-3 legs | PQ373245 | Jan. 2023 | -28.88,31.46 | Luomus |
| *A. concolor* | Entumeni | South Africa | Entu17 | 2-3 legs | PQ373246 | Jan. 2023 | -28.88,31.46 | Luomus |
| *A. concolor* | Entumeni | South Africa | Entu18 | 2-3 legs | PQ373247 | Jan. 2023 | -28.88,31.46 | Luomus |
| *A. concolor* | Entumeni | South Africa | Entu19 | 2-3 legs | PQ373248 | Jan. 2023 | -28.88,31.46 | Luomus |
| *A. concolor* | Entumeni | South Africa | Entu20 | 2-3 legs | PQ373249 | Jan. 2023 | -28.88,31.46 | Luomus |
| *A. concolor* | Nkandla | South Africa | Nkan1 | caterpillar | PQ373250 | Jan. 2023 | -28.62,31.09 | Luomus |
| *A. concolor* | Nkandla | South Africa | Nkan2 | caterpillar | PQ373251 | Jan. 2023 | -28.62,31.09 | Luomus |
| *A. concolor* | Nkandla | South Africa | Nkan3 | caterpillar | PQ373252 | Jan. 2023 | -28.62,31.09 | Luomus |
| *A. concolor* | Nkandla | South Africa | Nkan_3.2 | caterpillar | PQ373253 | Jan. 2023 | -28.62,31.09 | Luomus |
| *A. concolor* | Nkandla | South Africa | Nkan4 | caterpillar | PQ373254 | Jan. 2023 | -28.62,31.09 | Luomus |
| *A. concolor* | Nkandla | South Africa | Nkan_4.2 | caterpillar | PQ373255 | Jan. 2023 | -28.62,31.09 | Luomus |
| *A. concolor* | Nkandla | South Africa | Nkan5 | caterpillar | PQ373256 | Jan. 2023 | -28.62,31.09 | Luomus |
| *A. concolor* | Nkandla | South Africa | Nkan6 | caterpillar | PQ373257 | Jan. 2023 | -28.62,31.09 | Luomus |
| *A. concolor* | Nkandla | South Africa | Nkan_6.2 | caterpillar | PQ373258 | Jan. 2023 | -28.62,31.09 | Luomus |
| *A. concolor* | Nkandla | South Africa | Nkan7 | caterpillar | PQ373259 | Jan. 2023 | -28.62,31.09 | Luomus |
| *A. concolor* | Nkandla | South Africa | Nkan_7.2 | caterpillar | PQ373260 | Jan. 2023 | -28.62,31.09 | Luomus |
| *A. concolor* | Nkandla | South Africa | Nkan8 | caterpillar | PQ373261 | Jan. 2023 | -28.62,31.09 | Luomus |
| *A. concolor* | Nkandla | South Africa | Nkan9 | caterpillar | PQ373262 | Jan. 2023 | -28.62,31.09 | Luomus |
| *A. concolor* | Nkandla | South Africa | Nkan_9.2 | caterpillar | PQ373263 | Jan. 2023 | -28.62,31.09 | Luomus |
| *A. concolor* | Nkandla | South Africa | Nkan10 | caterpillar | PQ373264 | Jan. 2023 | -28.62,31.09 | Luomus |
| *A. concolor* | Nkandla | South Africa | Nkan_10.2 | caterpillar | PQ373265 | Jan. 2023 | -28.62,31.09 | Luomus |
| *A. concolor* | Nkandla | South Africa | Nkan_11 | caterpillar | PQ373266 | Jan. 2023 | -28.62,31.09 | Luomus |
| *A. concolor* | Nkandla | South Africa | Nkan_11_1 | caterpillar | PQ373267 | Jan. 2023 | -28.62,31.09 | Luomus |
| *A. concolor* | Nkandla | South Africa | Nkan13 | caterpillar | PQ373268 | Jan. 2023 | -28.62,31.09 | Luomus |
| *A. concolor* | Nkandla | South Africa | Nkan14 | caterpillar | PQ373269 | Jan. 2023 | -28.62,31.09 | Luomus |
| *A. concolor* | Ngoye | South Africa | Ngoy1 | caterpillar | PQ373270 | Jan. 2023 | -28.84,31.73 | Luomus |
| *A. concolor* | Ngoye | South Africa | Ngoy2 | caterpillar | PQ373271 | Jan. 2023 | -28.84,31.73 | Luomus |
| *A. concolor* | Ngoye | South Africa | Ngoy3 | caterpillar | PQ373272 | Jan. 2023 | -28.84,31.73 | Luomus |
| *A. concolor* | Ngoye | South Africa | Ngoy4 | caterpillar | PQ373273 | Jan. 2023 | -28.84,31.73 | Luomus |
| *A. concolor* | Ngoye | South Africa | Ngoy5 | caterpillar | PQ373274 | Jan. 2023 | -28.84,31.73 | Luomus |
| *A. concolor* | Ngoye | South Africa | Ngoy_5_1 | caterpillar | PQ373275 | Jan. 2023 | -28.84,31.73 | Luomus |
| *A. concolor* | Ngoye | South Africa | Ngoy6 | caterpillar | PQ373276 | Jan. 2023 | -28.84,31.73 | Luomus |
| *A. concolor* | Ngoye | South Africa | Ngoy7 | caterpillar | PQ373277 | Jan. 2023 | -28.84,31.73 | Luomus |
| *A. concolor* | Ngoye | South Africa | Ngoy10 | caterpillar | PQ373278 | Jan. 2023 | -28.84,31.73 | Luomus |
| *A. concolor* | Ngoye | South Africa | Ngoy_10_1 | caterpillar | PQ373279 | Jan. 2023 | -28.84,31.73 | Luomus |
| *A. concolor* | Ngoye | South Africa | Ngoy12 | caterpillar | PQ373280 | Jan. 2023 | -28.84,31.73 | Luomus |
| *A. concolor* | Ngoye | South Africa | Ngoy15 | caterpillar | PQ373281 | Jan. 2023 | -28.84,31.73 | Luomus |
| *A. concolor* | Ngoye | South Africa | Ngoy16 | caterpillar | PQ373282 | Jan. 2023 | -28.84,31.73 | Luomus |
| *A. concolor* | Ngoye | South Africa | Ngoy_16_1 | caterpillar | PQ373283 | Jan. 2023 | -28.84,31.73 | Luomus |
| *A. concolor* | Pennington | South Africa | Penn1 | caterpillar | PQ373284 | Jan. 2023 | -30.38,30.70 | Luomus |
| *A. concolor* | Pennington | South Africa | Penn2 | caterpillar | PQ373285 | Jan. 2023 | -30.38,30.70 | Luomus |
| *A. concolor* | Pennington | South Africa | Penn3 | caterpillar | PQ373286 | Jan. 2023 | -30.38,30.70 | Luomus |
| *A. concolor* | Pennington | South Africa | Penn4 | caterpillar | PQ373287 | Jan. 2023 | -30.38,30.70 | Luomus |
| *A. concolor* | Pennington | South Africa | Penn5 | caterpillar | PQ373288 | Jan. 2023 | -30.38,30.70 | Luomus |
| *A. concolor* | Pennington | South Africa | Penn8 | caterpillar | PQ373289 | Jan. 2023 | -30.38,30.70 | Luomus |
| *A. concolor* | Pennington | South Africa | Penn9 | caterpillar | PQ373290 | Jan. 2023 | -30.38,30.70 | Luomus |
| *A. concolor* | Pennington | South Africa | Penn10 | caterpillar | PQ373291 | Jan. 2023 | -30.38,30.70 | Luomus |
| *A. concolor* | Pennington | South Africa | Penn11 | caterpillar | PQ373292 | Jan. 2023 | -30.38,30.70 | Luomus |
| *A. concolor* | Pennington | South Africa | Penn12 | caterpillar | PQ373293 | Jan. 2023 | -30.38,30.70 | Luomus |
| *A. concolor* | Pennington | South Africa | Penn13 | caterpillar | PQ373294 | Jan. 2023 | -30.38,30.70 | Luomus |
| *A. concolor* | Pennington | South Africa | Penn14 | caterpillar | PQ373295 | Jan. 2023 | -30.38,30.70 | Luomus |

**Table S2.** ddRAD data summary

| **Sample ID** | **total reads** | **reads passed filter** | **clusters total** | **avg depth total** | **hetero est** | **error est** | **reads consens** | **heterozygosity** | **loci in assembly** |
| --- | --- | --- | --- | --- | --- | --- | --- | --- | --- |
| GSH13 | 560764 | 560747 | 7267 | 47.41 | 0.00224 | 0.00062 | 6394 | 0.0008 | 108 |
| GSH24 | 556420 | 556396 | 4941 | 67.31 | 0.00228 | 0.00050 | 4356 | 0.0007 | 71 |
| GSH27.2 | 94309 | 94307 | 2248 | 33.17 | 0.00088 | 0.00049 | 2015 | 0.0005 | 30 |
| GSH34.2 | 529947 | 529932 | 6374 | 50.91 | 0.00141 | 0.00050 | 5550 | 0.0005 | 91 |
| Kiba2 | 793785 | 793764 | 35201 | 19.34 | 0.00783 | 0.00071 | 30467 | 0.0034 | 738 |
| Kiba3 | 587770 | 587759 | 34059 | 13.7 | 0.00637 | 0.00081 | 30076 | 0.0020 | 615 |
| Kiba8.2 | 376090 | 376078 | 8000 | 23.25 | 0.00151 | 0.00067 | 5834 | 0.0006 | 120 |
| Kiba9.2 | 1224898 | 1224873 | 7546 | 43.47 | 0.00454 | 0.00099 | 4062 | 0.0009 | 60 |
| Kiba10.2 | 306584 | 306574 | 5290 | 35.59 | 0.00214 | 0.00088 | 4098 | 0.0009 | 118 |
| Kiba11.2 | 960581 | 960544 | 8319 | 24.78 | 0.00204 | 0.00076 | 6627 | 0.0007 | 123 |
| Kuhe1 | 918707 | 918668 | 39810 | 11.53 | 0.00732 | 0.00062 | 26485 | 0.0018 | 4982 |
| Kuhe1_1 | 1203819 | 1203775 | 43912 | 12.36 | 0.00711 | 0.00065 | 29516 | 0.0020 | 5300 |
| Kuhe1_2 | 2329170 | 2329111 | 97561 | 12.67 | 0.00673 | 0.00066 | 58179 | 0.0013 | 5478 |
| Kuhe2 | 791214 | 791185 | 19385 | 20.61 | 0.00479 | 0.00076 | 11805 | 0.0006 | 1438 |
| Kuhe2_1 | 1249169 | 1249137 | 41179 | 9.27 | 0.00785 | 0.00088 | 26565 | 0.0012 | 3809 |
| Kuhe3 | 471838 | 471828 | 36614 | 5.95 | 0.00952 | 0.00200 | 20419 | 0.0017 | 3601 |
| Kuhe3_1 | 946472 | 946444 | 52958 | 8.24 | 0.00735 | 0.00070 | 33469 | 0.0015 | 4901 |
| Kuhe3_2 | 2363333 | 2363265 | 50179 | 18.94 | 0.00635 | 0.00058 | 33610 | 0.0021 | 5566 |
| Kuhe4.2 | 544432 | 544416 | 29152 | 8.57 | 0.00617 | 0.00065 | 20152 | 0.0020 | 4731 |
| Kuhe4_1.2 | 1027060 | 1027040 | 31734 | 12.41 | 0.00668 | 0.00059 | 22455 | 0.0020 | 5015 |
| Kuhe5 | 1082337 | 1082294 | 48130 | 10.36 | 0.00680 | 0.00069 | 31077 | 0.0017 | 5085 |
| Kuhe5_1 | 1055951 | 1055916 | 39473 | 12.12 | 0.00666 | 0.00066 | 25402 | 0.0016 | 4502 |
| MtAl2 | 1712798 | 1712748 | 63334 | 14.92 | 0.00950 | 0.00183 | 50762 | 0.0022 | 5147 |
| MtAl3 | 379813 | 379801 | 20170 | 9.02 | 0.00548 | 0.00111 | 15468 | 0.0012 | 2427 |
| MtAl4 | 800276 | 800252 | 37622 | 10.28 | 0.00715 | 0.00071 | 30608 | 0.0020 | 4974 |
| MtAl5 | 790022 | 790003 | 27528 | 11.89 | 0.00521 | 0.00083 | 21534 | 0.0015 | 3675 |
| MtAl6 | 1548505 | 1548462 | 43205 | 14.35 | 0.00676 | 0.00076 | 35417 | 0.0020 | 5408 |
| MtAl9 | 2520054 | 2519982 | 68786 | 17.35 | 0.00627 | 0.00063 | 56831 | 0.0018 | 5896 |
| MtAl12 | 847285 | 847258 | 31775 | 12.52 | 0.00714 | 0.00084 | 26598 | 0.0022 | 5194 |
| MtAl13 | 1007232 | 1007213 | 24365 | 11.97 | 0.00506 | 0.00067 | 20093 | 0.0017 | 4143 |
| MtAl14 | 1097859 | 1097834 | 37024 | 13.69 | 0.00690 | 0.00077 | 30935 | 0.0022 | 5334 |
| MtAl15 | 831124 | 831097 | 33126 | 11.08 | 0.00652 | 0.00078 | 27939 | 0.0018 | 4886 |
| MtAl17 | 797199 | 797178 | 28181 | 12.12 | 0.00748 | 0.00070 | 23370 | 0.0021 | 4770 |
| StLu1 | 1403866 | 1403821 | 49460 | 12.54 | 0.00791 | 0.00073 | 37041 | 0.0017 | 4889 |
| Dlin1 | 1007142 | 1007117 | 43723 | 15.89 | 0.00263 | 0.00052 | 35934 | 0.0006 | 10752 |
| Dlin2 | 582662 | 582647 | 25500 | 15.21 | 0.00275 | 0.00051 | 21760 | 0.0007 | 9656 |
| Dlin4 | 1027083 | 1027059 | 28368 | 16.66 | 0.00334 | 0.00056 | 22319 | 0.0007 | 6386 |
| Dlin5 | 603723 | 603704 | 24913 | 12.95 | 0.00273 | 0.00058 | 20313 | 0.0006 | 7715 |
| Dlin6 | 1262256 | 1262226 | 50935 | 16.89 | 0.00248 | 0.00057 | 41579 | 0.0006 | 10812 |
| Dlin7 | 1178294 | 1178263 | 41538 | 19.02 | 0.00265 | 0.00054 | 34429 | 0.0007 | 10794 |
| Dlin8 | 970129 | 970104 | 41089 | 16.07 | 0.00267 | 0.00059 | 33781 | 0.0007 | 10358 |
| Dlin9 | 2071922 | 2071860 | 74000 | 17.86 | 0.00283 | 0.00049 | 59639 | 0.0006 | 10770 |
| Dlin10 | 734262 | 734247 | 37832 | 11.71 | 0.00337 | 0.00078 | 28183 | 0.0007 | 9562 |
| Dlin12 | 738025 | 738009 | 48682 | 9.52 | 0.00391 | 0.00070 | 35146 | 0.0006 | 9691 |
| Dlin14 | 683049 | 683023 | 36317 | 10.39 | 0.00333 | 0.00067 | 26880 | 0.0007 | 9413 |
| Dlin15 | 441210 | 441194 | 35676 | 7.51 | 0.00358 | 0.00068 | 24575 | 0.0006 | 7384 |
| Entu2 | 896640 | 896622 | 42910 | 14.62 | 0.00237 | 0.00056 | 35672 | 0.0006 | 10370 |
| Entu3 | 660119 | 660101 | 31824 | 13.64 | 0.00279 | 0.00064 | 26590 | 0.0006 | 9363 |
| Entu4 | 1407081 | 1407038 | 43199 | 19.45 | 0.00324 | 0.00048 | 35786 | 0.0007 | 10721 |
| Entu9 | 301911 | 301903 | 19437 | 11.05 | 0.00199 | 0.00051 | 15780 | 0.0006 | 6706 |
| Entu12 | 658957 | 658943 | 31595 | 13.78 | 0.00301 | 0.00059 | 24266 | 0.0007 | 9985 |
| Entu14 | 1627290 | 1627240 | 64531 | 15.99 | 0.00235 | 0.00060 | 47607 | 0.0005 | 10213 |
| Entu15 | 1228897 | 1228861 | 57177 | 13.59 | 0.00242 | 0.00064 | 38576 | 0.0006 | 10564 |
| Entu16 | 1152266 | 1152244 | 54198 | 14.06 | 0.00249 | 0.00056 | 40432 | 0.0006 | 10764 |
| Entu17 | 1709322 | 1709270 | 58878 | 17.58 | 0.00229 | 0.00059 | 43418 | 0.0006 | 10827 |
| Entu18 | 891389 | 891372 | 45774 | 12.51 | 0.00247 | 0.00067 | 34059 | 0.0005 | 9176 |
| Entu19 | 1114571 | 1114544 | 55006 | 13.58 | 0.00249 | 0.00059 | 40062 | 0.0005 | 10336 |
| Entu20 | 1741614 | 1741567 | 57458 | 18.52 | 0.00320 | 0.00048 | 42053 | 0.0006 | 11001 |
| Nkan2 | 1090073 | 1090042 | 39826 | 7.86 | 0.00311 | 0.00067 | 25310 | 0.0006 | 8395 |
| Nkan3.2 | 1790314 | 1790262 | 108252 | 10.7 | 0.00281 | 0.00045 | 64454 | 0.0004 | 10739 |
| Nkan4.2 | 1001784 | 1001742 | 66804 | 10.15 | 0.00324 | 0.00053 | 41489 | 0.0006 | 10964 |
| Nkan5 | 618377 | 618357 | 47829 | 8.82 | 0.00327 | 0.00069 | 31569 | 0.0006 | 10291 |
| Nkan6.2 | 927017 | 926984 | 58137 | 10.78 | 0.00248 | 0.00067 | 38221 | 0.0006 | 10778 |
| Nkan7.2 | 1218058 | 1218017 | 66898 | 11.5 | 0.00303 | 0.00064 | 42819 | 0.0006 | 10960 |
| Nkan9.2 | 1950991 | 1950923 | 95792 | 12.57 | 0.00234 | 0.00059 | 58647 | 0.0005 | 11027 |
| Nkan10.2 | 1222450 | 1222404 | 40517 | 19.8 | 0.00277 | 0.00054 | 29069 | 0.0008 | 11329 |
| Nkan11 | 1403006 | 1402961 | 71258 | 11.78 | 0.00322 | 0.00060 | 44956 | 0.0005 | 10973 |
| Nkan11_1 | 1172171 | 1172130 | 56628 | 12.45 | 0.00337 | 0.00055 | 36316 | 0.0006 | 10396 |
| Nkan13 | 836652 | 836618 | 56547 | 8.97 | 0.00422 | 0.00070 | 35477 | 0.0005 | 8691 |
| Ngoy1 | 706517 | 706486 | 48785 | 9.74 | 0.00387 | 0.00076 | 31747 | 0.0007 | 10057 |
| Ngoy2 | 1433031 | 1432975 | 43730 | 20.61 | 0.00236 | 0.00056 | 39440 | 0.0006 | 10431 |
| Ngoy3 | 1400144 | 1400109 | 31988 | 17.16 | 0.00323 | 0.00073 | 27960 | 0.0007 | 7085 |
| Ngoy5 | 2010058 | 2009996 | 33905 | 18.28 | 0.00258 | 0.00071 | 30312 | 0.0006 | 9379 |
| Ngoy5_1 | 1467926 | 1467891 | 38697 | 25.98 | 0.00231 | 0.00067 | 35154 | 0.0007 | 10943 |
| Ngoy6 | 776934 | 776913 | 22462 | 15.91 | 0.00284 | 0.00080 | 19962 | 0.0006 | 6416 |
| Ngoy7 | 520264 | 520247 | 15140 | 20.05 | 0.00158 | 0.00059 | 13457 | 0.0006 | 5302 |
| Ngoy10_1 | 732676 | 732658 | 30909 | 15.37 | 0.00265 | 0.00079 | 26564 | 0.0006 | 9005 |
| Ngoy12 | 578056 | 578036 | 21581 | 17.87 | 0.00282 | 0.00067 | 19424 | 0.0006 | 8132 |
| Ngoy15 | 1215511 | 1215462 | 37549 | 15.58 | 0.00362 | 0.00103 | 31857 | 0.0007 | 8534 |
| Ngoy16_1 | 637762 | 637744 | 24661 | 17.54 | 0.00236 | 0.00071 | 22546 | 0.0006 | 9400 |
| Penn1 | 1798275 | 1798219 | 46278 | 26.09 | 0.00224 | 0.00052 | 42061 | 0.0007 | 10916 |
| Penn2 | 1119778 | 1119744 | 24973 | 10.48 | 0.00809 | 0.00144 | 17918 | 0.0010 | 5778 |
| Penn3 | 781121 | 781097 | 42452 | 9.49 | 0.00707 | 0.00135 | 30175 | 0.0014 | 7833 |
| Penn4 | 1234834 | 1234803 | 155339 | 3.94 | 0.01611 | 0.00142 | 30922 | 0.0016 | 8640 |
| Penn5 | 1055134 | 1055096 | 39550 | 15.62 | 0.00329 | 0.00072 | 31350 | 0.0007 | 9386 |
| Penn8 | 922225 | 922186 | 35914 | 15.43 | 0.00311 | 0.00062 | 28438 | 0.0007 | 9388 |
| Penn9 | 151708 | 151707 | 12140 | 7.38 | 0.00210 | 0.00054 | 9198 | 0.0005 | 2923 |
| Penn10 | 2098430 | 2098352 | 62368 | 17.08 | 0.00350 | 0.00065 | 47311 | 0.0007 | 10120 |
| Penn11 | 1536843 | 1536807 | 33695 | 23.54 | 0.00359 | 0.00076 | 25845 | 0.0009 | 9347 |
| Penn12 | 688589 | 688564 | 22634 | 13.69 | 0.00245 | 0.00060 | 17492 | 0.0006 | 6587 |
| Penn13 | 1452234 | 1452184 | 68829 | 14.24 | 0.00280 | 0.00056 | 52264 | 0.0006 | 10215 |
| Penn14 | 469566 | 469548 | 20917 | 11.68 | 0.00239 | 0.00062 | 15726 | 0.0006 | 6240 |
| **Average** | **1048033** | **1048001** | **41168** | **16.19** | **0.00417** | **0.00071** | **29560** | **0.0010** | **7099** |

**Table S3.** Different species delimitation models for the group evaluated with the BFD* method and their results. Each row indicates different species scenarios. The best scenario is shown in bold. Abbreviations refer to each population: kiba1, kiba2, SA_North (kuhe, MtAl, StLu), SA_South (Entu, Dlin, Nkan, Ngoy, Penn).

| **Scenarios** | **Description** | **Species number** | **MLE** | **BF** | **Rank** |
| --- | --- | --- | --- | --- | --- |
| 2 sp | Kiba1 and the rest of populations merged | 2 | -6729.022 | 2155.3 | 10 |
| 3 sp | Kiba1, Kiba2, and the rest of populations | 3 | -6024.848 | 746.9 | 9 |
| **4 sp** | **Kiba1, Kiba2, SA_North, SA_South** | **4** | **-5651.384** | **0** | **1** |
| 2ks | Kiba1 + Kiba2, SA_North + SA_South | 2 | -5757.948 | 213.1 | 2 |
| 3ns | Kiba1 + Kiba2, SA_North, SA_South | 3 | -5766.481 | 230.2 | 3 |
| 5 sp | Kiba1, Kiba2, Kuhe, MtAl+StLu, SA_South | 5 | -5814.931 | 327.1 | 8 |
| 6 sp | Kiba1, Kiba2, Kuhe, MtAl+StLu, Entu+Dlin+Nkan+Ngoy, Penn | 6 | -5768.852 | 234.9 | 4 |
| 7 sp | Kiba1, Kiba2, Kuhe, MtAl+StLu, Entu+Dlin+Nkan, Ngoy, Penn | 7 | -5773.150 | 243.5 | 5 |
| 8 sp | Kiba1, Kiba2, Kuhe, MtAl+StLu, Entu+Dlin, Nkan, Ngoy, Penn | 8 | -5783.826 | 264.9 | 6 |
| 10 sp | All populations separated, except MtAl+StLu | 10 | -5808.611 | 314.5 | 7 |

Kiba, Kibale; Kuhe, Kuhestan; MtAl, Mt. Aloe Den; StLu, St. Lucia; Entu, Entumeni; Dlin, Dlinza; Nkan, Nkandla; Ngoy, Ngoye; Penn, Pennington.


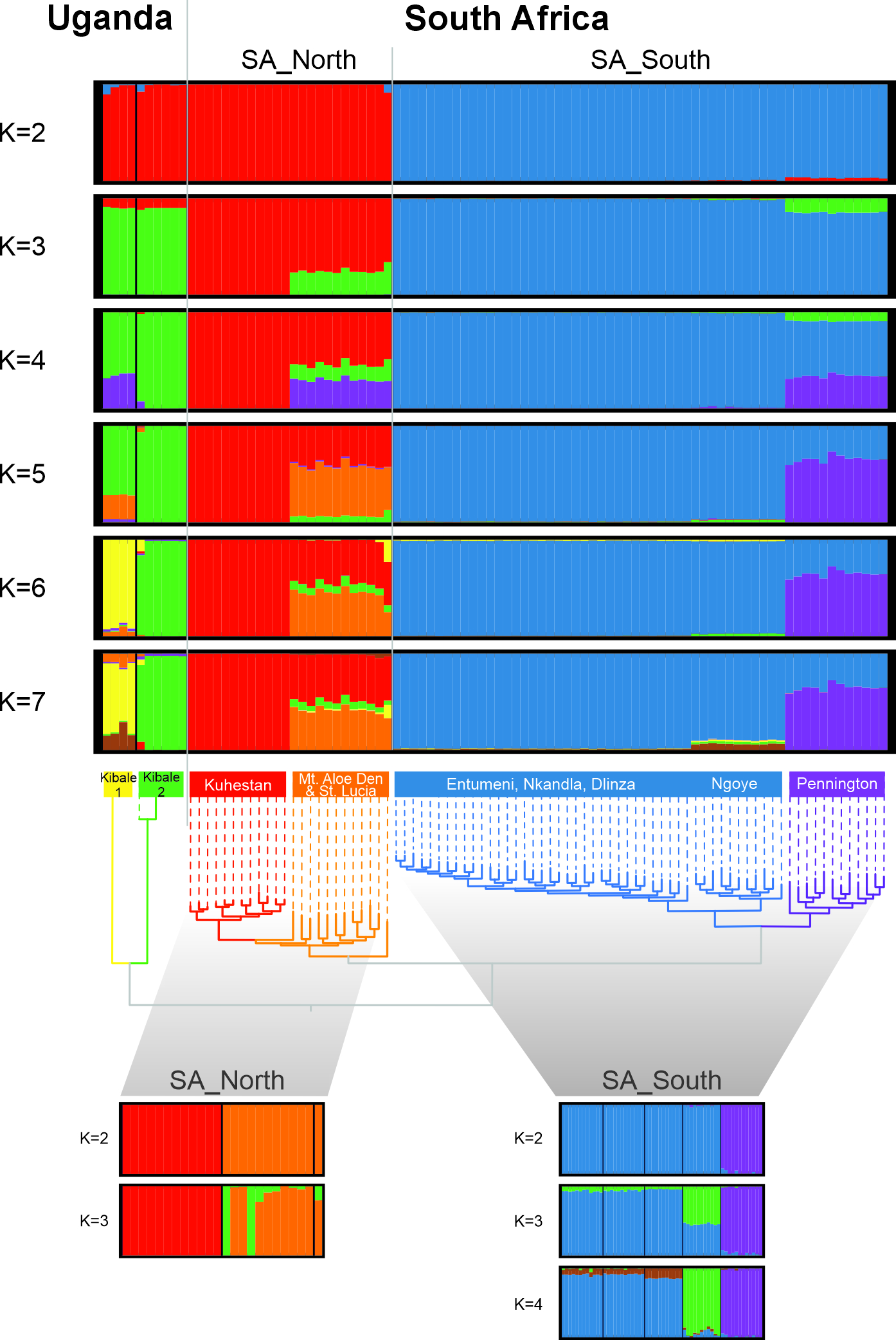


| # K | Reps | Mean LnP(K) | Stdev LnP(K) | Ln'(K) | \|Ln''(K)\| | Delta K |
| --- | --- | --- | --- | --- | --- | --- |
| 1 | 10 | -290160 | 13.41 | NA | NA | NA |
| 2 | 10 | -176825 | 97.27 | 113335.39 | 739658 | 7604.28 |
| 3 | 10 | -803147 | 428132.31 | -626322.64 | 656414 | 1.53 |
| 4 | 10 | -2085884 | 3978421.37 | -1282736.53 | 5E+06 | 1.25 |
| 5 | 10 | -8327813 | 4278716.26 | -6241929.09 | 1E+07 | 2.63 |
| **6** | **10** | **-3323915** | **500000.01** | **5003898.08** | **5E+07** | **100** |
| 7 | 10 | -6236820 | 4984189.75 | -2912905.14 | 6E+06 | 1.18 |
| 8 | 10 | -3283235 | 3715430.57 | 2953584.84 | 8E+06 | 2.1 |
| 9 | 10 | -8130159 | 7945150.64 | -4846923.97 | 7E+06 | 0.86 |
| 10 | 10 | -6164276 | 6351159.45 | 1965883.44 | NA | NA |

**Fig. S1.** STRUCTURE plots generated using different values of estimated clusters (K=2 to 7). This plot indicates ancestry proportions from K inferred genetic groups. Summary of mean LnP(K), standard deviation, and delta K values were calculated following Evanno et al. (2005).


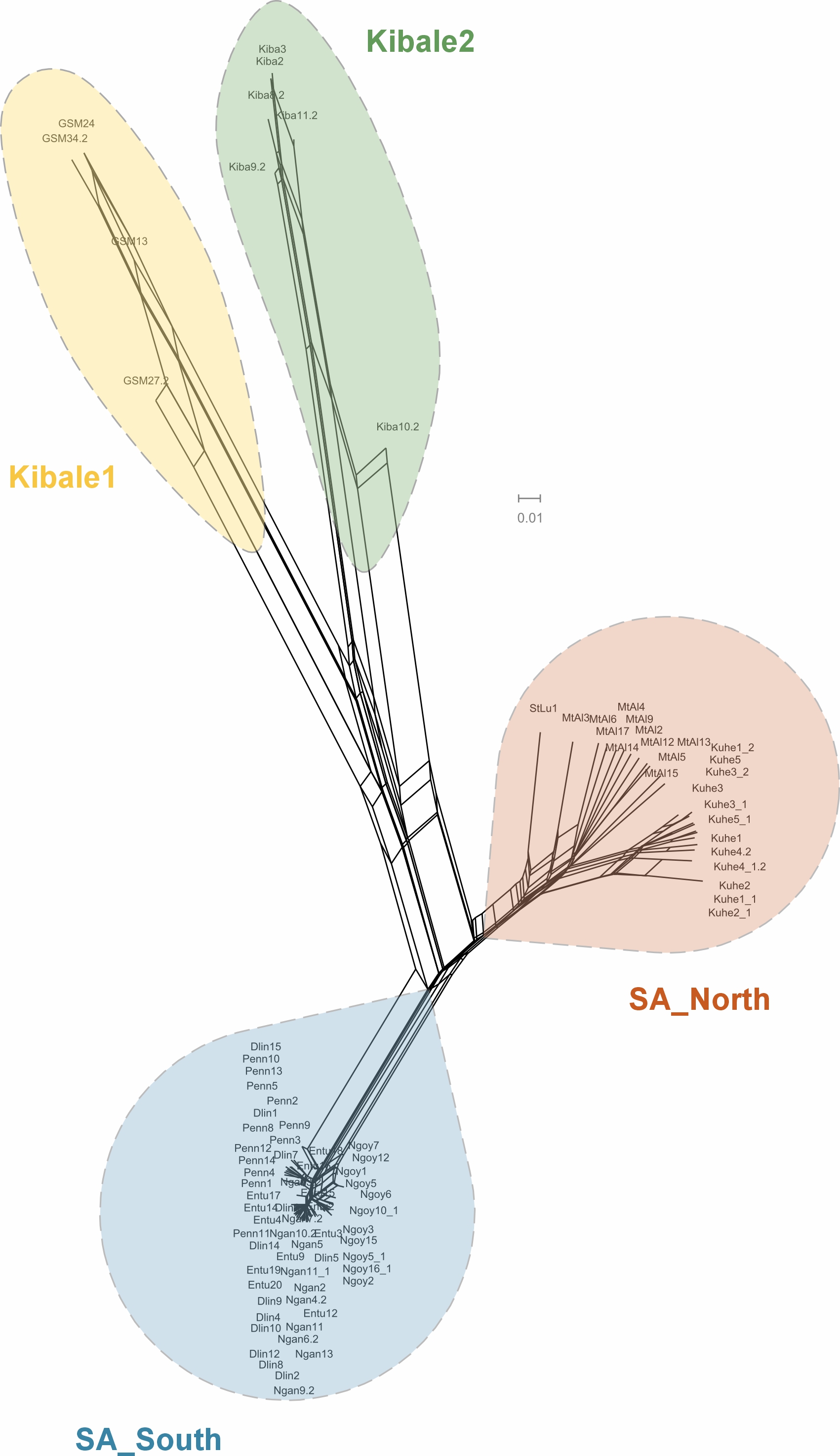


**Fig. S2.** SplitsTree built using uncorrelated P distances based on SNP data.
